# Lessons from a small country about the global obesity crisis

**DOI:** 10.1186/1744-8603-2-11

**Published:** 2006-09-12

**Authors:** Kelly D Brownell, Derek Yach

**Affiliations:** 1 Professor of Psychology, Epidemiology and Public Health, Director, Rudd Center for Food Policy and Obesity, Yale University – Rudd Center, 309 Edwards Street – Box 208369, New Haven, CT 06520–8369, USA; 2 Director Global Health, Rockefeller Foundation, New York, USA

## Abstract

Developed countries had high obesity rates before the problem was taken seriously and hence the genesis must be seen in retrospect. Developing countries offer a clear view of causal factors but also opportunities for prevention, which must focus on both food and physical activity environments.

## 

Statistics from country after country show increasing prevalence of obesity, with extreme prevalence in some areas. A cascade of diseases follows from overnutrition, inactivity, and obesity, including major killers such as heart disease, cancer, and diabetes. These chronic diseases were once the worry of the developed world but are now a chief health concern in developing countries. Figure 1 shows the alarming increases in diabetes expected in developed vs. developing countries [1].

**Figure 1:**
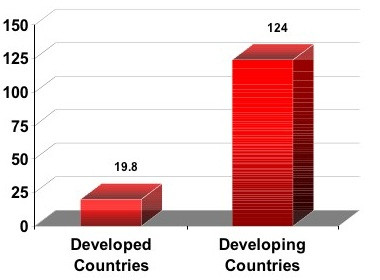


One can be numb to obesity statistics, but it is difficult to ignore 88% of adults of Kosrae, one of four districts in Micronesia, being overweight and 53% being obese. Such tragedies are magnified if we ignore their lessons. Cassels [2] offers an excellent analysis of the Kosrae experience, noting that "...the recent development of a wage-based economy has upset traditional eating habits and made them dependent on imported food from developed countries. Whereas previously individuals relied heavily on local foods such as fresh tuna, increasingly men and women use cash to buy nutrient poor packaged food." A major shift occurred in 1993 when the Federated States of Micronesia sold fishing rights to Japan.

Globalization changes many features of modern life, including diets. As trade changes, diets can become more secure (hunger becomes less of a problem), but the cheapening of calories, the reliance on imported food, and the influence of food marketing drive up consumption and drive down nutrient density. Obesity, diabetes, and other chronic diseases are not far behind. Fully 11% of global trade is in food [3]. Its impact is so profound that it has changed the relationship between income and fat consumption. Whereas fat intake was once higher in those with higher incomes, the reverse is now true [4].

One hopes there is still time for countries to see this coming and take preventive action, but history offers a depressing picture. Smoking is a key example. It took America decades to mobilize after the catastrophic consequences of smoking were clear, but when it did, American tobacco companies exploited overseas markets, particularly in the developing world. The tobacco industry in countries like China saw the potential as well and hence smoking rates, like rates of obesity, have been skyrocketing in China and in countries such as Indonesia, Botswana, and Uruguay. By the year 2025 the number of smokers worldwide is expected to increase by 45% and by 2030 the deaths attributed to smoking will increase from 4 to 10 million. The epidemic simply migrates from one part of the world to another.

Must development and obesity be close kin? Thus far the answer is yes. With development comes: 1) highly processed, energy-dense food from multinational companies that cheapens calories; 2) growth opportunities for the food industry to market foods and beverages with highest profits margins in developing countries; 3) less physical labor needed to raise and secure food; and 4) the rise of service-based economies and technological advances that further erode physical activity.

Only fundamental changes in thinking can uncouple this relationship. A first step is to understand what truly causes obesity. It is de rigueur to say a combination of genetics, environment, and psychosocial factors is at work, but this describes almost all human conditions and becomes a paralyzing excuse for inaction. Genetic differences between groups may set upper and lower limits on obesity, but whether an obesity crisis unfolds is clearly the impact of environment. To define the key environmental contributors offers up possibilities for prevention.

A number of specific actions are possible. The underlying principle is to change conditions such that healthy choices become the default. Among them are:

1) Support local farmers and promote the consumption of local foods.

2) Regulate the marketing of unhealthy food, particularly to children. This is justified by a large body of evidence.

3) Teach media literacy to buffer individuals against food marketing.

4) Discourage consumption of categories of foods known to contribute to poor diet and obesity, beginning where the science is strongest – soft drinks [5].

5) Focus on food norms that emphasize food quality and nutrient density over quantity and price.

6) Monitor and modify economic conditions that support (if not subsidize) unhealthy over healthy foods and create conditions where healthy foods are the logical economic choice.

7) Protect opportunities and incentives for physical activity.

## Competing interests

The author(s) declare that they have no competing interests.
